# A Simplified 3D Model of Whole Heart Electrical Activity and 12-Lead ECG Generation

**DOI:** 10.1155/2013/134208

**Published:** 2013-04-22

**Authors:** Siniša Sovilj, Ratko Magjarević, Nigel H. Lovell, Socrates Dokos

**Affiliations:** ^1^University of Zagreb, Faculty of Electrical Engineering and Computing, 10000 Zagreb, Croatia; ^2^Graduate School of Biomedical Engineering, University of New South Wales, Sydney, NSW 2052, Australia

## Abstract

We present a computationally efficient three-dimensional bidomain model of torso-embedded whole heart electrical activity, with spontaneous initiation of activation in the sinoatrial node, incorporating a specialized conduction system with heterogeneous action potential morphologies throughout the heart. The simplified geometry incorporates the whole heart as a volume source, with heart cavities, lungs, and torso as passive volume conductors. We placed four surface electrodes at the limbs of the torso: *V*_*R*_, *V*_*L*_, *V*_*F*_ and *V*_GND_ and six electrodes on the chest to simulate the Einthoven, Goldberger-augmented and precordial leads of a standard 12-lead system. By placing additional seven electrodes at the appropriate torso positions, we were also able to calculate the vectorcardiogram of the Frank lead system. Themodel was able to simulate realistic electrocardiogram (ECG) morphologies for the 12 standard leads, orthogonal *X*, *Y*, and *Z* leads, as well as the vectorcardiogram under normal and pathological heart states. Thus, simplified and easy replicable 3D cardiac bidomain model offers a compromise between computational load and model complexity and can be used as an investigative tool to adjust cell, tissue, and whole heart properties, such as setting ischemic lesions or regions of myocardial infarction, to readily investigate their effects on whole ECG morphology.

## 1. Introduction

Biophysically based mathematical models of whole-heart electrical activity are becoming increasingly detailed and complex, with high-resolution anatomically accurate models requiring extensive computation times, dedicated software, and even the use of supercomputers [1–3]. We have developed a simplified, computationally highly efficient three-dimensional (3D) torso-embedded whole heart model, capable of reproducing realistic 12-lead surface electrocardiograms (ECGs) on the torso. The model incorporates spontaneous activation in the natural pacemaker of the heart, the sinoatrial node (SAN), as well as the His-Purkinje specialized conduction system. Also included are heterogeneous action potential (AP) morphologies in various regions of the heart, whose parameters such as duration and amplitude can be readily adjusted to simulate their influence on ECG waveforms.

This simplified whole heart model can be utilized as a starting point for the rapid a priori formulation, testing, and refinement of hypothesis on the forward and inverse relations between the surface ECG and cardiac cell or tissue properties, including action potential duration, refractory period, upstroke velocity, amplitude, conduction velocity, and tissue conductivity in various subregions of the heart. This will allow the ready visualization of the relation between specific myocardial electrical properties and the resulting body surface potentials, expressed as a standard 12-lead ECG system, a vectorcardiogram (VCG), an electrogram (EGM), a body surface potential map (BSPM), or any other lead system. Such a model could readily be utilized to simulate regions of the heart with reduced excitability and/or conduction disturbance, for the development of new diagnostic tools able to inversely assess key cardiac properties or localization of ischemic or infarcted regions by processing features of the ECG.

It has been suggested that it may not be necessary to use overly complex cardiac models to gain insights into cardiac electrical activity and ECG characteristics; simplified models can be developed first and progressively expanded to more complex and detailed models [4].

To date, there has been a lack of a general purpose, easy replicable, and simplified 2D or 3D model of the torso-embedded heart [5, 6], perhaps because simplified models are considered unable to generate realistic ECG morphologies, despite the fact that such simplified models have been mainly used for cardiac tissue modeling [1, 2]. However, simplified models are easy to replicate, offering a good compromise between computational load and the electrophysiological complexity. If possible, they should be considered as an important stepping stone towards anatomically and functionally more detailed computer models of cardiac electrical activity [4, 7].

## 2. Methods and Materials

**Figure 1:**
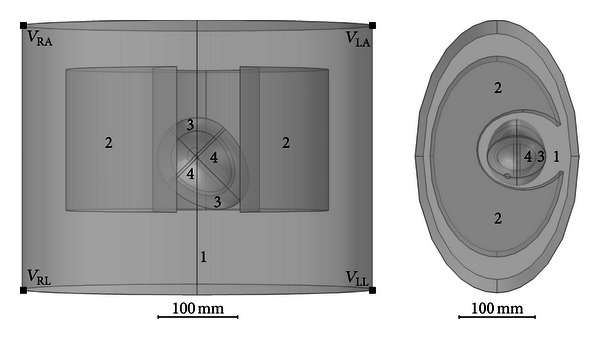
3D geometry of the model with subdomains labelled as follows: 1 = torso, 2 = lungs, 3 = atria and ventricles, and 4 = cardiac blood chambers, observed in the frontal plane (left) and the transverse plane (right).

The cardiac torso bidomain model developed in this study comprised a simplified 3D description of the torso, lungs, and the whole heart including atria, ventricles, and blood chambers, as shown in Figure 1. The torso and lungs were extruded from ellipses, with a cavity inserted into the lung domains for positioning the heart. Dimensions of all shapes were approximations to real human anatomy from the Visible Human Project gallery. All model domains have an assigned extracellular conductivity to account for volume conductor effects (Table 1), with values assigned from the literature [8, 9].

**Figure 2:**
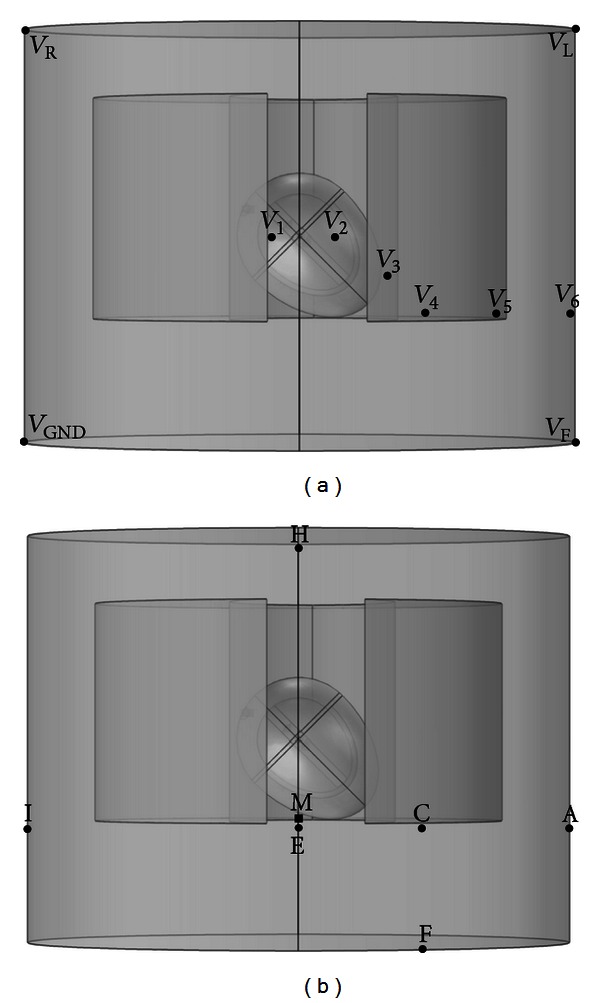
Frontal views of model showing placement of all ECG leads. (a) ECG electrodes are placed at the four corners of the torso, yielding surface potentials (*V*) of the right arm (*V*_*R*_), the left arm (*V*_*L*_), the left leg (*V*_*F*_), and the right leg (*V*_GND_). Additional six precordial leads, *V*_1_, *V*_2_, *V*_3_, *V*_4_, *V*_5_,  and  *V*_6_, were placed at the chest near the heart. (b) The seven electrodes (*A*, *C*, *E*, *F*, *H*, *I* anteriorly, and *M* posteriorly) are placed to form a Frank lead system for determining orthogonal *X*, *Y*, and *Z* components for the vectorcardiogram.

Modelling of the standard Einthoven and Goldberger-augmented leads was achieved using four ECG electrodes at the vertices of the torso corresponding to positions of the left arm (*V*_*L*_), right arm (*V*_*R*_), left foot (*V*_*F*_), and right leg (*V*_GND_), as shown in Figure 2(a). Additional six electrodes (*V*_1_,  *V*_2_,  *V*_3_,  *V*_4_,  *V*_5_, and  *V*_6_) were positioned at the chest to model the precordial leads, completing all channels of a 12-lead ECG system. Furthermore, seven additional electrodes (*A*,  *C*,  *E*,  *I*,  *F*,  *H*,  and  *M*) were placed on the surface of the chest (Figure 2(b)) to determine the VCG using a Frank lead system. The right leg electrode (*V*_GND_) was set as the ground reference, so the potential at that node was fixed at 0 V.

The governing equation for the extracellular voltage *V* in the passive volume conductor regions (excluding the myocardium) was given by the Laplace formulation

(1) $$\begin{array}{c} \nabla \cdot \left(-\sigma_{o}\nabla V\right)=0, \end{array}$$

where *σ*_*o*_ is the electrical conductivity of respective outside heart subdomain: torso, lungs, and cardiac blood chambers, with values given in Table 1. All exterior boundaries of the torso were set to be electrically insulating (zero normal component of current density), and all the interior boundaries in contact with the heart were set to *V* = *V*_*e*_ where *V*_*e*_ is the extracellular voltage in the myocardial walls.

The three Einthoven leads *V*_I_, *V*_II_, and *V*_III_ from the standard 12-lead ECG were determined from

(2) $$\begin{array}{c} V_{\text{I}}=V_{L}-V_{R},\\ V_{\text{II}}=V_{F}-V_{R},\\ V_{\text{III}}=V_{F}-V_{L}. \end{array}$$

The other leads were obtained directly from electrodes on the torso from the six precordial leads *V*_1_,  *V*_2_,  *V*_3_,  *V*_4_,  *V*_5_, and *V*_6_ (Figure 2(a)) or by implementing additional calculations: the three Goldberger-augmented leads were calculated from

(3) $$\begin{array}{c} aV_{R}=\frac{\left(2V_{R}-V_{L}-V_{F}\right)}{2},\\ aV_{L}=\frac{\left(2V_{L}-V_{R}-V_{F}\right)}{2},\\ aV_{F}=\frac{\left(2V_{F}-V_{R}-V_{L}\right)}{2}, \end{array}$$

while the precordial leads were calculated as differences of each precordial electrode from the Wilson central terminal *V*_*CT*_ = (*V*_*L*_ + *V*_*R*_ + *V*_*F*_)/3. Furthermore, the orthogonal leads *X*,*Y*, and *Z* for the VCG were determined from the Frank lead system by placing additional six electrodes *A*, *C*, *E*, *F*, *H*, and *I* at the anterior surface of the torso and additional electrode *M* at the posterior (Figure 2(b)). The orthogonal leads were computed as

(4) $$\begin{array}{c} X=0.610A+0.171C-0.781I,\\ Y=0.655F+0.345M-1.000H,\\ Z=0.133A+0.736M-0.264I-0.374E-0.231C \end{array}$$

in accordance with existing methods [10, 11]. Moreover, with an additional calculation, the root mean square curve can be derived as $V_{\text{RMS}}=\sqrt{{(V}_{\text{I}}^{2}+V_{\text{II}}^{2}+V_{\text{III}}^{2})/3}$, and a similar equation can be used for the signal averaged ECG (SAECG), $V_{\text{SAECG}}=\sqrt{\left(X^{2}+Y^{2}+Z^{2}\right)/3}$.

**Figure 3:**
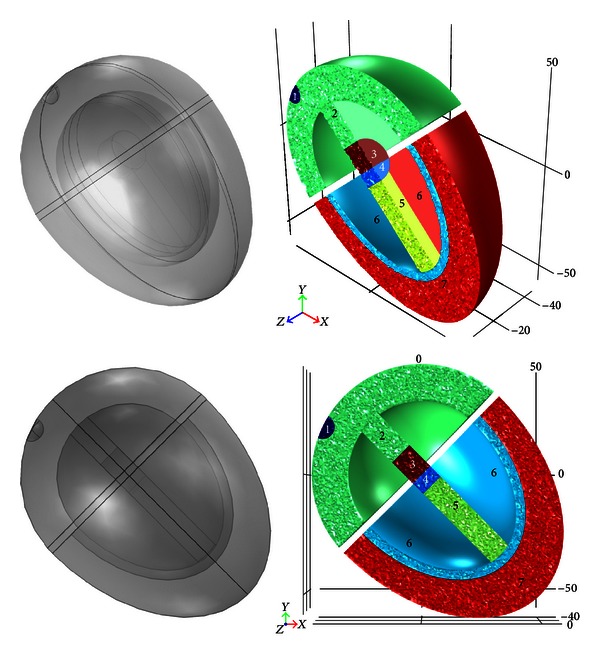
Whole heart layout with semitransparent views in the right panels and sectioned views illustrating the various subdomains of the heart in panels on the right. Subdomain numbering is as follows: 1 = sinoatrial node, 2 = atria, 3 = atrioventricular node, 4 = His bundle, 5 = bundle branches, 6 = Purkinje fibers, and 7 = ventricular myocardium.

The heart itself was divided into seven subdomains or regions with heterogeneous cardiac cell properties and tissue conductivities representing specialized cells of the conduction system and the myocardium (Figure 3). An electrical isolation gap exists between the atria and ventricles except at a junction in the septum that links the atrioventricular node (AVN) with the His bundle.

The bidomain model of cellular activation of the heart, including the SAN, was defined at the cellular level by three dependent variables: *V*_*e*_: the extracellular potential, *V*_*i*_: the intracellular potential, and *u*: a recovery variable governing cellular refractoriness. The bidomain equations were based on modified FitzHugh-Nagumo equations [12, 13]. For each region of the heart, they were defined according to

(5) $$\begin{array}{c} \frac{\partial V_{e}}{\partial t}-\frac{\partial V_{i}}{\partial t}+\nabla \cdot \left(-\sigma_{e}\nabla V_{e}\right)=i_{\text{ion}},\\ \frac{\partial V_{i}}{\partial t}-\frac{\partial V_{e}}{\partial t}+\nabla \cdot \left(-\sigma_{i}\nabla V_{i}\right)=-i_{\text{ion}},\\ \frac{\partial u}{\partial t}=ke\left[\frac{\left(V_{m}-B\right)}{A}-du-b\right] \end{array}$$

with *σ*_*e*_, *σ*_*i*_ denoting the extracellular and intracellular conductivities, respectively, *V*_*m*_ = *V*_*i*_ − *V*_*e*_, and *a*, *b*, *c*_1_, *c*_2_, *d*,*e*, *k*, *A*, and *B* are region-specific parameters, while *i*_ion_ is defined according to

(6) $$\begin{array}{c} i_{\text{ion}}=kc_{1}\left(V_{m}-B\right)\left[a-\frac{\left(V_{m}-B\right)}{A}\right]\left[1-\frac{\left(V_{m}-B\right)}{A}\right]+kc_{2}u \end{array}$$

within the SAN and

(7) iion=kc1(Vm−B)[a−(Vm−B)A][1−(Vm−B)A]+kc2u(Vm−B)

within the walls of the atria, ventricles, AVN, His bundle, bundle branches, and Purkinje fibers.

Parameters of the model region-specific values are listed in Table 2. The parameter *e* mainly regulates action potential duration (APD), whilst conductivity parameters *σ*_*e*_, *σ*_*i*_ control conduction velocities in the tissue which are well known from the literature [9]. For example, a lower conductivity in the AVN performs an appropriate delay in impulse conduction to the ventricles. The initial values of all model variables are given in Table 3.

Boundary conditions on all interior boundaries in contact with the torso, lungs, and cardiac cavities are zero flux for *V*_*i*_; therefore, −**n** · Γ = 0 where **n** is the unit outward normal vector on the boundary and Γ is the flux vector through that boundary for the intracellular voltage, equal to Γ = −*σ*_*i*_ · ∂*V*_*i*_/∂**n**. For the variable *V*_*e*_, the inward flux on these boundaries is equal to the outward current density **J** from the torso/chamber volume conductor; therefore, −*σ*_*e*_ · ∂*V*_*e*_/∂**n** = **n** · **J**.

The simplified 3D cardiac model was simulated using COMSOL Multiphysics (COMSOL AB, Switzerland, v4.2a) finite element software. The resulting finite element mesh consisted of 21106 tetrahedral elements with 51680 degrees of freedom to be solved at each time step. Simulations took approximately 1 hour to solve one second of cardiac activity with 1 ms output time resolution. The simulations were performed on an Intel Core i7-970 PC workstation, with processing power of about 100 Gflops.

In order to validate the model against typical cardiomyopathies encountered in clinical practice, we simulated two myocardial infarcts (MIs) and their resulting effect on the ECG: (a) an anterior MI in the apical section of the heart and (b) an inferior MI. We also examined the possibility of localizing and identifying these two common infarction types with our model. For simulating the infarcts, the intracellular conductivity *σ*_*i*_ and parameter *k* were set to zero in the infarcted regions, with initial values for *V*_*i*_ and *V*_*e*_ set to −60 mV and −20 mV, respectively. Both types of infarct were positioned apically, spanning the epicardium, midmyocardium, and endocardium (Figure 7).

## 3. Results and Discussion

**Figure 4:**
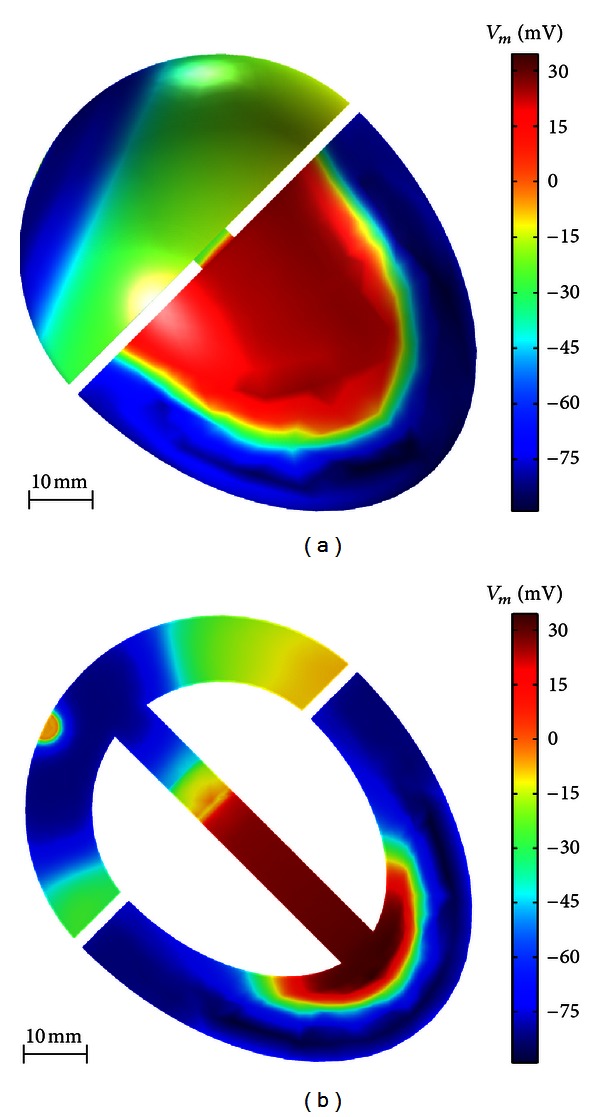
*V*_*m*_ transmembrane potential (TMP) at the heart surface (a) and in a frontal plane cross section midway through the heart (b), when the depolarization wave front first excites the left and right ventricles and when atrial repolarization has just begun.

In all simulations, spontaneous and periodic rhythmic activation occurred in the SAN pacemaker region, located in the right atrial wall of the heart. The electrical activation impulse then spread throughout the atria and through the atrial septum before reaching the AVN, where the excitation wavefront was delayed until the atria were entirely activated. Subsequently, the AVN activated the His bundle from where the activation spread to the bundle branches, the Purkinje fibers, and the whole ventricles (Figure 4).

**Figure 5:**
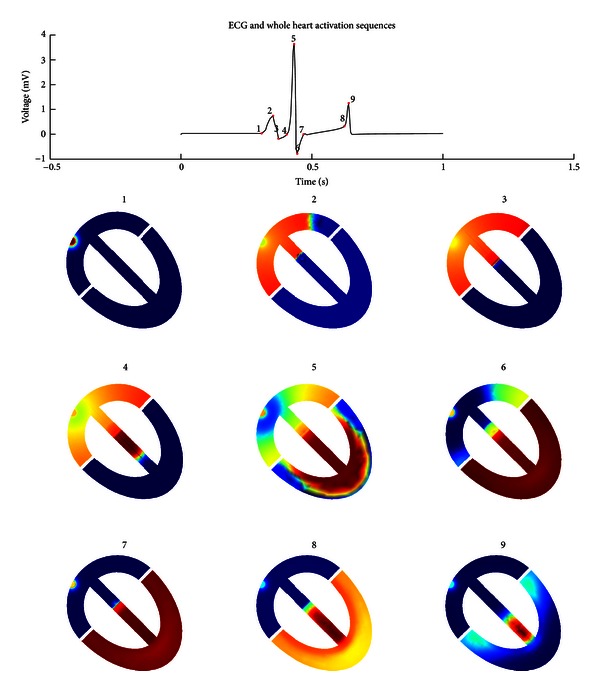
Simulated lead II ECG signal and corresponding whole heart activation sequences at various time points on the ECG signal. The numeric labels on the ECG mark the moments in which the matching activation sequences below are illustrated. The color bar at Figure 4 also applies for the current figure.

Figure 5 illustrates the relationship between the Einthoven lead II ECG and the state of excitation in the heart in a frontal plane cross section, in which characteristic time-points are labeled on the ECG with the matching activation states of the heart. The activation sequences demonstrate the progression of depolarization and repolarization wavefronts, showing that the AVN imposes a time delay until the whole atria are activated in sequence 3, whilst in sequences 5 and 6 there is an overlap between ventricular depolarization and atrial repolarization. The peak of the *P* wave (sequence 2) is seen to occur when most of the atria have been depolarized, whilst the QRS complex (sequences 4, 5, and 6) corresponds to the activation of the His and Purkinje regions and the entire depolarization of the ventricles. The peak of the *T* wave (sequence 9) occurs when both ventricles are fully in their repolarization phase.

**Figure 6:**
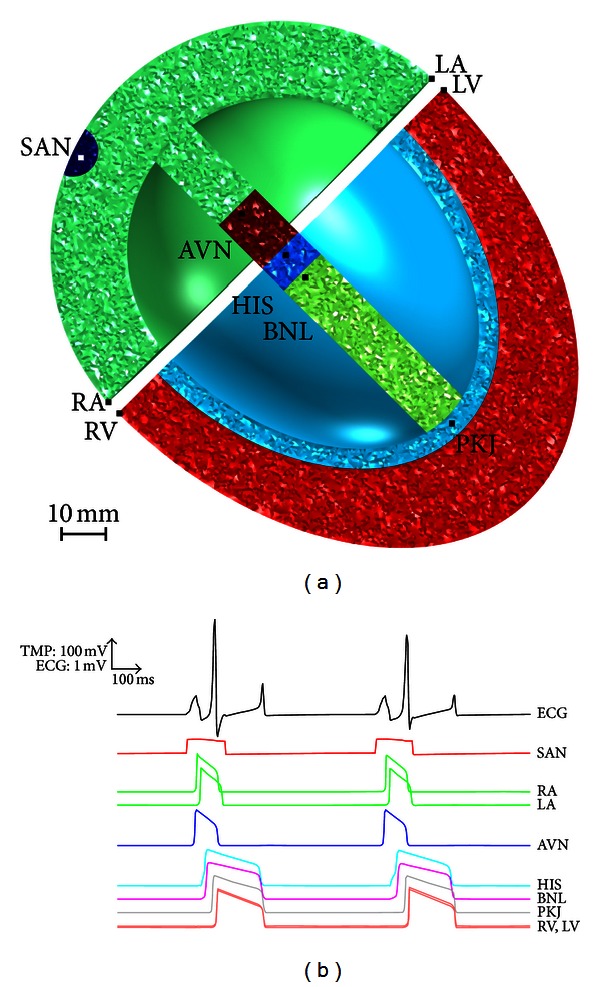
Simulation of normal electrical activity. (a) Frontal plane cross section midway through the heart with the probe locations (black dots) positioned throughout the myocardium according to sinoatrial node (SAN), right atria (RA), left atria (LA), atrioventricular node (AVN), His bundle (HIS), bundle branches (BNL), Purkinje fibers (PKJ), right ventricle (RV), and left ventricle (LV). (b) Simulated lead II ECG waveform and the transmembrane action potentials at the probe positions.

Figure 6 illustrates the time course of the simulated ECG and the transmembrane action potentials (TMPs) at various myocardial locations over an interval of 2 seconds. As noted, the excitation in the SAN is spontaneous, stable, and periodic. The ECG morphology, action potential durations, and activation times throughout the heart agree well with known behavior.

**Figure 7:**
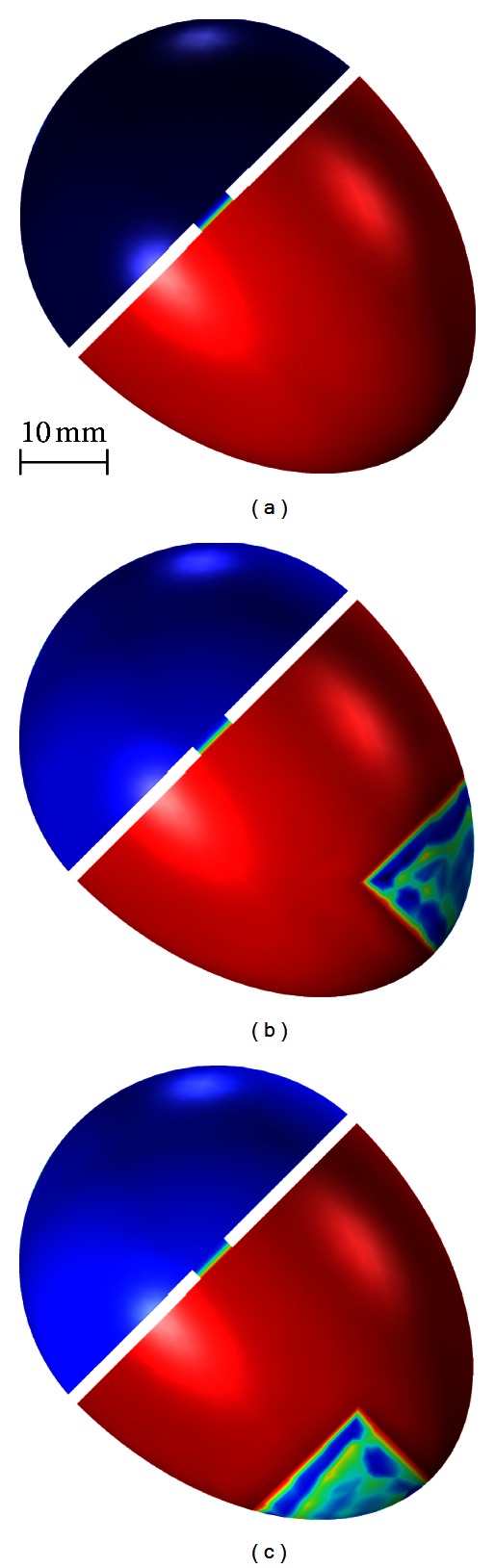
Simulated transmembrane potential *V*_*m*_ at the epicardial surface in normal heart (a), heart with anterior MI (b), and heart with inferior MI (c).

Figure 7 illustrates the transmembrane potential *V*_*m*_ at the epicardial surface when the ventricles are completely depolarized for the three cases: (1) normal heart, (2) heart with anterior myocardial infarction (anterior MI), and (3) heart with inferior myocardial infarction (inferior MI). The simulations indicate the absence of activation in both types of infarct.

**Figure 8:**
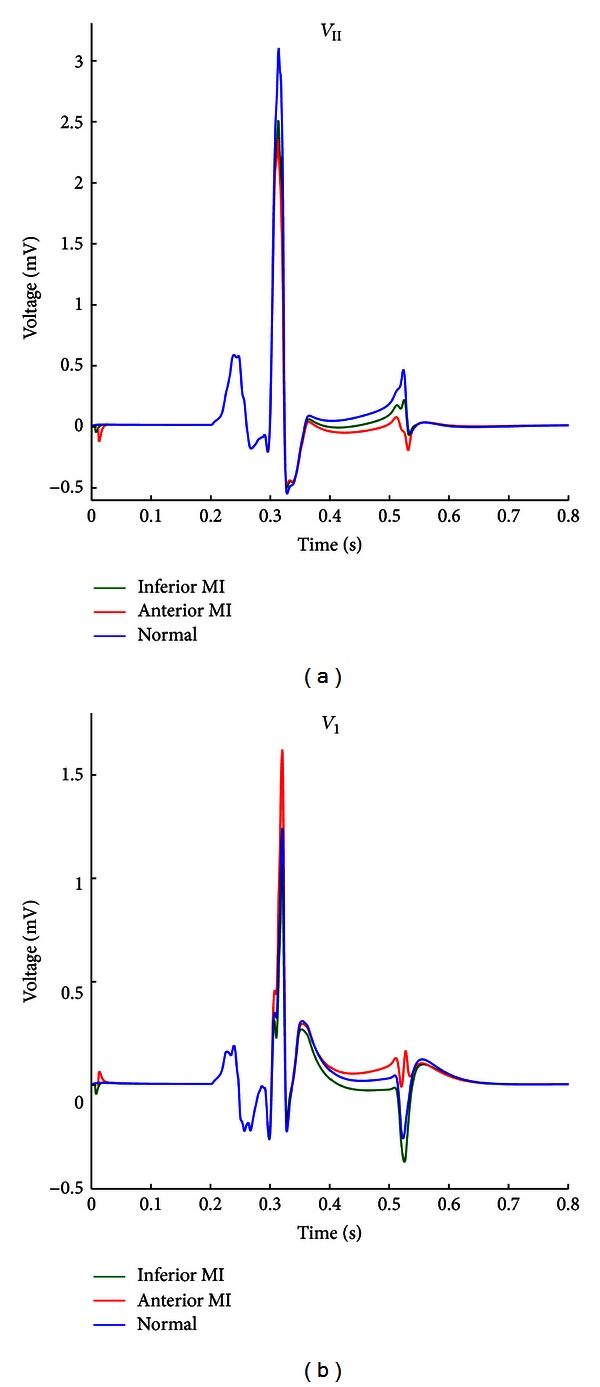
Simulated Lead II ECG (a) and precordial lead *V*_1_ (b) for the three cases: normal heart (blue), anterior MI (red), and inferior MI (green).

Figure 8 shows the simulated ECG morphology in both lead II and precordial lead *V*_1_ for the three cases shown in Figure 7. The lead II waveform reveals ST segment depression (which starts at the *J* point and ends at the beginning of the *T* wave) for both infarct types, with the anterior MI exhibiting deeper ST segment depression than the inferior type (Figure 1, left). The difference between anterior and inferior MI is even more pronounced in precordial lead *V*_1_ (Figure 8(b)), since the former leads to ST segment elevation and the latter exhibits depression. These findings of ST segment depression or elevation match their clinical usage as a diagnostic criterion for detection of myocardial infarction [14], whilst MI localization from this criterion is reported to be not very accurate [15]. It is important to emphasize that the exact heart angle as well as the position and orientation of the septum within the torso substantially determines the level of ST segment change. Simplified 3D cardiac models, such as the one described in this study, could be useful as a basis for the refinement and enhancement of ECG-based methods for detection and localization of myocardial infarctions.

**Figure 9:**
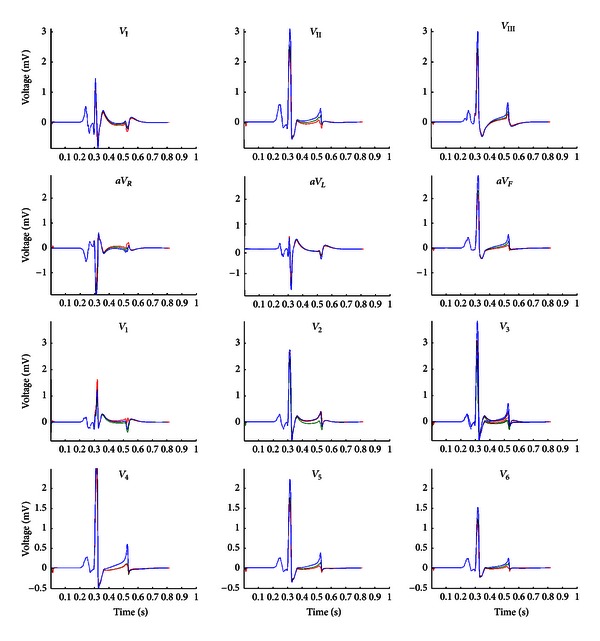
Simulated 12 ECG leads for three cases: normal heart (blue), heart with anterior MI (red), and heart with inferior MI (green). *V*_I_, *V*_II_, and *V*_III_ refer to the three Einthoven leads; *aV*_*R*_, *aV*_*L*_, and *aV*_*F*_ are the augmented limb leads, whilst *V*_1_–*V*_6_ denote the six precordial leads.

Figure 9 illustrates the simulated ECG for all 12 leads (Einthoven, Goldberger-augmented, and precordial leads) for the three cases considered above: normal heart, anterior MI, and inferior MI. For most of the ECG leads, the presence of myocardial infarction is disclosed as either ST segment elevation or depression relative to the normal ECG, in agreement with standard clinical findings [14].

**Figure 10:**
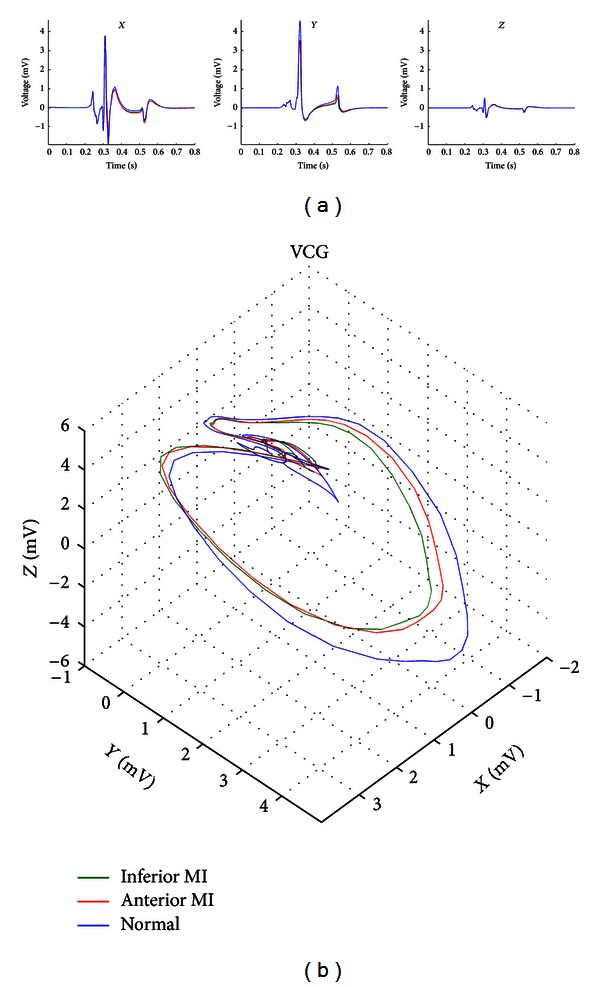
Simulated vectorcardiogram (VCG) (b) and *X*, *Y*, and *Z* orthogonal ECG leads (a) for three cases: normal heart (blue), heart with anterior MI (red), and heart with inferior MI (green).

Figure 10 illustrates the three simulated orthogonal leads ,*Y*, and *Z* along with reconstructed VCG for the same three cases: normal heart, anterior MI, and inferior MI. Although the ST segment depressions and elevations for the infarction cases are observable in all three orthogonal leads, it is not as easy to clearly identify these slight ST segment differences in the VCG.

## 4. Conclusion

We have developed a simplified 3D model of the torso-embedded whole heart capable of generating spontaneous and morphologically realistic 12-lead ECG signals at the torso surface, offering a good compromise between computational efficiency, model complexity, and simulated ECG signal quality. 

Two cases were simulated in which myocardial infarctions were imposed at different heart regions, and the impact on ECG morphology was observed. The simulation results showed a change in the ST segment level, confirming well-established current clinical diagnostic criteria for identification of myocardial infarction based on the ECG.

Our cardiac 3D model offers wide possibilities for modeling various cardiac myopathies and electrical abnormalities, in order to explore and develop new ECG-based diagnostic methods. By providing a good compromise between computational load and model complexity, the model can be used as a stepping stone towards anatomically more detailed models with realistic myocardial architecture, including anisotropic fiber conductivity.

In the future, the simplified 3D cardiac model could even be useful for inverse estimation, whereby cardiac electrical function could be assessed from body surface potentials by estimating model parameters from forward model solutions to obtain meaningful and unique reconstructions of electrical activity at the level of the heart.

## Figures and Tables

**Table 1:** Electrical conductivity values for model tissues.

Subdomain	Conductivity (S/m)	Conductivity (S/m)	Conductivity (S/m)
	[8]	[9]	Present study
Heart	0.054	0.400 (longitudinal) 0.179 (transversal)	0.05
Blood	0.7	0.625	0.7
Lungs	0.203 (deflated) 0.039 (inflated)	0.050	0.04
Muscle	0.202	0.526 (parallel) 0.076 (normal)	N/A
Fat	0.012	0.040	N/A
Bone	0.000975 (marrow)	0.006	N/A
Torso	N/A	N/A	0.2

**Table 2:** Model parameters by region.

Parameter	SAN	Atria	AVN	His	BNL	Purkinje	Ventricles
*a*	−0.60	0.13	0.13	0.13	0.13	0.13	0.13
*b*	−0.30	0	0	0	0	0	0
*c*_1_ (A·s·V^−1^ m^−3^)	1000	2.6	2.6	2.6	2.6	2.6	2.6
*c*_2_ (A·s·V^−1^ m^−3^)	1.0	1.0	1.0	1.0	1.0	1.0	1.0
*d*	0	1	1	1	1	1	1
*e*	0.0660	0.0132	0.0132	0.0050	0.0022	0.0047	0.0060
*A* (mV)	33	140	140	140	140	140	140
*B* (mV)	−22	−85	−85	−85	−85	−85	−85
*k* (s^−1^)	1000	1000	1000	1000	1000	1000	1000
*σ*_*e*_ (mS·m^−1^)	0.5	8	0.5	10	15	35	8
*σ*_*i*_ (mS·m^−1^)	0.5	8	0.5	10	15	35	8

**Table 3:** Initial variable values by region.

Variable	SAN	Atria	AVN	His	BNL	Purkinje	Ventricles
*V*_*i*_ (V)	−0.06	−0.085	−0.085	−0.085	−0.085	−0.085	−0.085
*V*_*e*_ (V)	0	0	0	0	0	0	0
*u*	0	0	0	0	0	0	0
